# Supplementary material for: Association between hemoglobin/red blood cell distribution width ratio and acute kidney injury in sepsis and heart failure patients
Source: PLoS One. 2026 Feb 27;21(2):e0331332. doi: 10.1371/journal.pone.0331332 (PMC12948117; doi:10.1371/journal.pone.0331332)
Supplement: S1 Table — AKI, acute kidney injury; S-AKI, sepsis-associated acute kidney injury; T1-CRS, Cardiorenal Syndrome type 1; CKD, chronic kidney disease; CRP, C Reactive Protein; NT-proBNP, N-terminal pro-B-type natriuretic peptide; Hb, hemoglobin concentration; MAP, mean arterial pressure; MCV, mean corpuscular volume; RDW, red blood cell distribution width; WBC, white blood cell; PaO2/FiO2, arterial oxygen pressure/inspired oxygen fraction; SAPS3, Simplified Acute Physiologic Score 3 prognostic index. (DOCX) [file pone.0331332.s001.docx]

**S1 Table.** Comparison of demographic and clinical data between sepsis-associated acute kidney injury and type 1-cardiorenal syndrome patients from intensive care unit admission.

|  | S-AKI (n=68) | T1-CRS (n=49) | | *p* |
| --- | --- | --- | --- | --- |
| Age (Years)  Male (%) | 78.2 ± 14.5  48 (70.6) | | 82.6 ± 8.7  31 (63.3) | 0.06  0.41 |
| Comorbidities, n (%)  *Diabetes mellitus*  *Hypertension*  *CKD*  *Smoker* | 30 (44.2)  37 (54.4)  6 (8.8)  10 (17.7) | 17 (34.7)  28 (57.1)  16 (32.6)  14 (28.6) | | 0.31  0.77  0.001  0.07 |
| MAP (mmHg) | 75.9±19.3 | 78.1±20.5 | | 0.57 |
| Creatinine (mg/dl) | 1.56±0.3 | 1.67±0.4 | | 0.18 |
| Urea (mg/dl) | 84.1±37.4 | 89.6±38.2 | | 0.43 |
| Sodium (mEq/l) | 135±17.3 | 135±5.2 | | 0.91 |
| Potassium (mEq/l) | 4.02±0.6 | 4.58±0.9 | | 0.001 |
| CRP (mg/l) | 128±13.8 | 131±15.7 | | 0.90 |
| Glycemia (mg/dl) | 147±67 | 134±59 | | 0.27 |
| Total bilirubin (mg/dl) | 1.3±0.2 | 0.95±0.1 | | 0.11 |
| NT-proBNP (pg/ml) | 2685±1297 | 2937±2118 | | 0.41 |
| Ejection fraction (%) | 61.2±9.3 | 47.1±14.5 | | <0.001 |
| Hb (g/dl) | 11.8±1.6 | 10.7±2.1 | | 0.002 |
| MCV (fl) | 91.7±7.2 | 91.5±12.4 | | 0.87 |
| RDW (%)  Hb/RDW (g/dl) | 15.3±2.3  79.5±17.6 | 16.2±1.6  66.7±15.9 | | 0.02  <0.001 |
| WBC (10^3^/µl) | 12.7±8.9 | 13.1±5.2 | | 0.83 |
| Platelets (10^3^/µl) | 190±11.5 | 196±13.2 | | 0.76 |
| Lactate (mg/dl) | 23.1±2.1 | 17.1±1.6 | | 0.03 |
| PaO_2_/FiO_2_ (mmHg) | 327±14.3 | 301±17.2 | | 0.22 |
| SAPS3  Outcome for CKD-5 | 64.2±16.4  3 (2.5) | 68.1±9.5  9 (10.8) | | 0.14  0.031 |
|  |  |  | |  |

*AKI, acute kidney injury; S-AKI, sepsis-associated acute kidney injury*; *T1-CRS, Cardiorenal Syndrome type 1; CKD, chronic kidney disease; CRP, C Reactive Protein; NT-proBNP,* N*-*terminal*pro-B-type natriuretic peptide; Hb, hemoglobin concentration; MAP, mean arterial pressure; MCV, mean corpuscular volume; RDW, red blood cell distribution width; WBC, white blood cell; PaO_2_/FiO_2_, arterial oxygen pressure/inspired oxygen fraction; SAPS3, Simplified Acute Physiologic Score 3 prognostic index;* CKD-5 – Chronic Kidney Disease Stage 5*.*
