# Supplementary material for: Association between hemoglobin/red blood cell distribution width ratio and acute kidney injury in sepsis and heart failure patients
Source: PLoS One. 2026 Feb 27;21(2):e0331332. doi: 10.1371/journal.pone.0331332 (PMC12948117; doi:10.1371/journal.pone.0331332)
Supplement: Attachment — Submitted filename: Response to Reviewers.doc [file pone.0331332.s002.doc]

Petherson Mendonça dos Santos

Department of Medicine

Nephrology Division,

Rua Borges Lagoa 783

Zip code: 04038-901

São Paulo, Brazil

T	+55 (11) 5576-4848, Ext.2478
E-mail: pethersonmds@outlook.com

May 1^st^, 2025

*Elabbass Abdelmahmuod
Academic Editor
PLOS ONE*

São Paulo Brazil

Dear Elabbass Abdelmahmuod,

We have carefully revised our manuscript, ***"Association between Hemoglobin/Red Blood Cell Distribution Width Ratio and Acute Kidney Injury in Sepsis and Heart Failure Patients"*** (PONE-D-25-12369).

First, we sincerely thank you for your thoughtful feedback concerning our manuscript. We are glad you found merit in our work and truly appreciate your encouraging and constructive comments.

Following your advice, we have addressed all the points raised during the review process and added new information to the manuscript. Below, we provide a detailed point-by-point response to each comment:

**Editor’s Comments:**

**1- Expand the discussion on potential confounders and alternative explanations for the observed associations.**

We thank the editor for this important suggestion. Our discussion section has significantly expanded to address potential confounders and alternative explanations for the observed associations (pages 18-19). Specifically, we have added comprehensive paragraphs addressing age-related factors and their impact on both Hb/RDW ratio and AKI risk, the role of inflammation as a potential confounder, medication exposure effects on kidney function and hematological parameters, the impact of comorbidities on study outcomes, also oxidative stress as a standard pathway affecting both parameters and alternative explanations involving endothelial dysfunction.

We have included appropriate references to support these discussions (references 41-50).

**2 - Clarify adjustments made for inflammatory markers and preexisting conditions.**

In the Methods section, we have clarified our methodological approach for adjusting inflammatory markers and preexisting conditions (page 08). Now, we explicitly describe how C-reactive protein (CRP) was selected as the primary inflammatory marker, the rationale for CRP selection and its measurement timing, how preexisting conditions (diabetes mellitus, hypertension, chronic kidney disease) were systematically incorporated, the role of SAPS 3 prognostic index in capturing clinical variability, and our backward stepwise selection process for the multivariate model.

**3 - Provide confidence intervals for key statistical findings in the results section.**

As requested, we have added confidence intervals throughout the Results section for all key statistical findings. It includes comparative analyses between groups with confidence intervals for differences, odds ratios with 95% confidence intervals from multivariate analysis, relative risks with 95% confidence intervals for clinical outcomes, and mean differences with 95% confidence intervals for continuous variables.

All additions are marked in the revised manuscript, pages 12 to 15.

**4 - Discuss how findings compare to existing literature on AKI predictors**

We have significantly expanded our discussion to compare our findings with existing literature on AKI predictors (pages 17-18). We added a comprehensive section that compares the Hb/RDW ratio with traditional biomarkers and discusses their respective advantages and limitations. We particularly emphasize the practical benefits of using the Hb/RDW ratio, especially its potential in resource-limited settings. This section also contextualizes our findings within previous research on RDW as a predictor of clinical outcomes, discusses the emerging trend of using combined biomarkers for improved risk stratification, and addresses the specific implications for heart failure patients and cardio-renal interactions.

Additionally, we have added a new paragraph that further strengthens this comparison by highlighting how the Hb/RDW ratio retains its predictive value alongside established clinical parameters (SAPS 3 and serum urea levels) in our logistic regression model. This new section not only clarifies how the Hb/RDW ratio can be integrated into current risk stratification strategies, but also reinforces its practical relevance, aligning with emerging trends that favor the use of composite markers to enhance prognostication in critical care settings. We have ensured that all new additions are supported by up-to-date and relevant references. These citations were carefully selected to better contextualize our findings within the evolving landscape of AKI prediction research. We hope this strengthens the overall clarity and credibility of our results.

**5 - Revise minor language issues to improve readability and clarity.**

We thank the editor for the valuable suggestion to improve the clarity and readability of our manuscript. In response, we carefully revised the text throughout, focusing on sentence structure, terminology, and overall flow. Specifically, we restructured complex sentences in the Abstract (page 4), Methods (pages 6–7), and Results (pages 9–15). We also standardized terminology and improved consistency in reporting statistical results, particularly in Tables 1–3 and their corresponding descriptions. The clarity of correlations and statistical findings in the Results section (pages 11–15) was enhanced, and we refined the description of our analytical approach in the Methods (page 8). Additionally, we improved sentence structure and paragraph organization in the Discussion (pages 15–19) to better convey our findings and their implications. All changes were made without altering the scientific content or conclusions. We believe these revisions substantially improve the overall readability and presentation of the manuscript.

**Additional Journal Requirements:**

**1. Manuscript formatting**

**Response:**  We confirm that the manuscript has been formatted in full accordance with the PLOS ONE guidelines, using the provided templates. All sections were reviewed to ensure compliance with formatting requirements, including margins, line spacing, heading structure, and reference style.

**2. Data availability statement**

**Response:** We have updated our Data Availability Statement to comply with PLOS ONE’s open data policy. The data supporting the findings of this study are available on Figshare at the following DOI: 10.6084/m9.figshare.28200248. The dataset includes only de-identified patient information and is fully compliant with data protection regulations and the protocol approved by our local ethics committee. Currently, the dataset is accessible via the DOI link with limited visibility and will be made fully public upon formal publication, in accordance with Figshare’s embargo feature.

**3. Supplementary tables**

**Response:** As requested, we have removed the supplementary tables from the main manuscript and uploaded them separately as Supporting Information files. Legends for each file have been added after the references section in the manuscript.

**4. Reference list review**

**Response:** We have thoroughly reviewed our reference list to ensure it is complete and accurate. All references cited in the manuscript are included in the reference list, and vice versa. We have confirmed that no retracted papers are cited. Additionally, we have added several new references (38-50) to support the expanded discussion on AKI predictors and potential confounders.

These revisions have significantly strengthened the manuscript, and we believe we have addressed all of the editor's concerns. We hope that the revised manuscript now meets the standards for publication in *PLOS ONE*.

Thank you for your time and consideration. We look forward to your feedback.

Sincerely,
On behalf of all co-authors,
Petherson Mendonça dos Santos
Division of Nephrology, Federal University of São Paulo, Brazil
Email: pethersonmds@outlook.com
