# Supplementary material for: Association between hemoglobin/red blood cell distribution width ratio and acute kidney injury in sepsis and heart failure patients
Source: PLoS One. 2026 Feb 27;21(2):e0331332. doi: 10.1371/journal.pone.0331332 (PMC12948117; doi:10.1371/journal.pone.0331332)
Supplement: Attachment — Submitted filename: Response to Reviewers (3).doc [file pone.0331332.s003.doc]

Petherson Mendonça dos Santos

Department of Medicine

Nephrology Division,

Rua Borges Lagoa 783

Zip code: 04038-901

São Paulo, Brazil

T	+55 (11) 5576-4848, Ext.2478
E-mail: pethersonmds@outlook.com

November 10^th^, 2025

*Helen Howard
Staff Editor
PLOS ONE*

São Paulo, Brazil

Dear Helen Howard,

We have carefully revised our manuscript, ***"Association between Hemoglobin/Red Blood Cell Distribution Width Ratio and Acute Kidney Injury in Sepsis and Heart Failure Patients"*** (PONE-D-25-12369R1).

First, we sincerely thank you for your thoughtful feedback concerning our manuscript. We are glad you found merit in our work and truly appreciate your encouraging and constructive comments.

Following your advice, we have addressed all the points raised during the review process and added new information to the manuscript. Below, we provide a detailed point-by-point response to each comment:

**Editor’s Comments:**

**The Editorial team has assessed your manuscript and concerns were raised over the small sample size and the fact that the data are from 2017. In order for us to consider the manuscript further, as part of this revision, the sample size will need to be increased and more recent data will need to be included.**

We appreciate the editor’s concern regarding the 2017 dataset. Our study included all consecutive ICU admissions for sepsis or acute decompensated heart failure (ADHF) during that year, with complete laboratory and outcome data retrieved from a unified institutional database before changes in the hospital’s electronic record system. A considerable number of ICU deaths occurred within the first 48 hours of admission, making it impossible to evaluate kidney injury dynamics in these cases and justifying their exclusion from the analysis.

To strengthen the dataset and respond to the editor’s request for more recent information, we conducted a long-term follow-up extending to July 2024, assessing renal outcomes such as progression to dialysis-dependent stage V chronic kidney disease (CKD-V). This longitudinal extension expanded the temporal scope of the study, allowing us to examine both short-term (AKI) and long-term (CKD progression) kidney outcomes (page 6).

We were unable to include additional patient recruitment beyond 2017 because of ethical restrictions and structural changes in the hospital’s electronic data system. In addition, the SARS-CoV-2 pandemic profoundly altered ICU admission patterns, making it difficult to merge pre- and post-pandemic data in a methodologically consistent way. For these reasons, the dataset we present represents a complete and homogeneous annual cohort, now supported by validated longitudinal follow-up. We believe this design minimizes selection bias and provides clinically relevant insight into both acute and chronic renal outcomes. Finally, the prognostic question explored—the predictive value of the Hb/RDW ratio in critically ill patients—remains highly relevant today, as confirmed by several studies published between 2024 and 2025.

**Reviewer Comments**

**1 - I do not understand patient selection. Actually in a relevant hospital in 1 year few patients seems to have been enrolled, being potentially not consecutive and leading to selection bias**

We thank the reviewer for this important observation. We have clarified the screening and enrollment process in the revised Methods section. This study included all consecutive ICU admissions diagnosed with sepsis or acute decompensated heart failure (ADHF) during a fixed one-year period (January 1 to December 31, 2017). A total of 386 critically ill adults were initially screened. After applying strict predefined exclusion criteria (hematologic/oncologic diseases, CKD-5 on dialysis, cirrhosis, chronic viral infections, and death within the first 48 hours in the ICU), 202 patients were deemed eligible and included in the final cohort. This approach ensures a complete consecutive annual cohort, thereby minimizing selection bias. To further enhance transparency, Figure 1 has been updated to clearly display the flow of patient screening, exclusion, and inclusion with exact numbers and the explicit note that admissions were consecutive (page 10).
**2 - Primary and secondary end points should be clearly stated.**

We have clearly defined the study endpoints in the revised Methods section (pages 6-8). The primary endpoint was the occurrence of acute kidney injury (AKI) during the ICU stay, according to KDIGO criteria; AKI was selected as the primary outcome given its central role in the pathophysiology of our population (patients with sepsis or acute decompensated heart failure). The secondary endpoints included: (1) need for kidney replacement therapy, (2) requirement for red blood cell transfusion, (3) need for mechanical ventilation, and (4) mortality at day 28 and (5) long-term renal outcome defined as progression to dialysis-dependent stage V chronic kidney disease (CKD-V) during a 7-year follow-up.
**3 - Sample size calculation should be added.**

We appreciate the reviewer’s suggestion. As a proof-of-concept, hypothesis-generating observational study based on a complete annual cohort of all consecutive eligible ICU admissions, a traditional a priori sample size calculation was not applicable to our design. Instead, to evaluate the adequacy of our sample for multivariable analysis, we adhered to the established methodological principle of at least 10 outcome events per candidate predictor variable. With 117 AKI events, our dataset would support up to 11 candidate predictors; in practice, only four variables were entered into the initial model and three were retained in the final model. This yielded an events-per-variable ratio well above recommended thresholds, ensuring statistical robustness and minimizing the risk of overfitting. Accordingly, the sample size is appropriate for the analytical approach adopted in this exploratory context.
All additions are marked in the revised manuscript, page 8.

**4 - It is not clear how variables were included in the multivariate analysis. In particular, a lot of variables (3 on 4) were significant potentially leading to low accuracy of the model.**

We thank the reviewer for this insightful comment. We have now clarified the variable selection process in the revised Statistical Analysis section. All variables showing an association with AKI at p < 0.10 in univariate analyses (serum urea at admission, SAPS 3, Hb/RDW ratio, and C-reactive protein [CRP]) were entered into a multivariable logistic regression model using a backward stepwise elimination approach (removal threshold p ≥ 0.10). This process was conducted while adhering to the methodological principle of at least 10 outcome events per candidate predictor (117 AKI events in total). The final parsimonious model retained serum urea, SAPS 3, and the Hb/RDW ratio, with CRP being removed during the stepwise selection process. The model demonstrated satisfactory discrimination (AUROC = 0.763) and adequate calibration (Hosmer–Lemeshow χ² = 8.283; p = 0.406). These revisions clarify the criteria for variable inclusion, demonstrate that overfitting was minimized, and confirm that the resulting model achieved appropriate accuracy and internal validity.All corresponding additions and clarifications are marked in the revised manuscript (page 13)

These revisions have significantly strengthened the manuscript, and we believe we have addressed all of the editor's concerns. We hope that the revised manuscript now meets the standards for publication in *PLOS ONE*.

Thank you for your time and consideration. We look forward to your feedback.

Sincerely,
On behalf of all co-authors,
Petherson Mendonça dos Santos
Division of Nephrology, Federal University of São Paulo, Brazil
Email: pethersonmds@outlook.com
